# Estrogen Receptors Alpha and Beta in Acute Myeloid Leukemia

**DOI:** 10.3390/cancers12040907

**Published:** 2020-04-08

**Authors:** Alessia Roma, Paul A. Spagnuolo

**Affiliations:** Department of Food Science, University of Guelph, 50 Stone Rd., Guelph, ON N1G2W1, Canada; aroma@uoguelph.ca

**Keywords:** estrogens, estrogen receptors (ERs), acute myeloid leukemia (AML), diosmetin, genistein, quercetin

## Abstract

Estrogen receptor (ER) signaling has been widely studied in a variety of solid tumors, where the differential expression of ERα and ERβ subtypes can impact prognosis. ER signaling has only recently emerged as a target of interest in acute myeloid leukemia (AML), an aggressive hematological malignancy with sub-optimal therapeutic options and poor clinical outcomes. In a variety of tumors, ERα activation has proliferative effects, while ERβ targeting results in cell senescence or death. Aberrant ER expression and hypermethylation have been characterized in AML, making ER targeting in this disease of great interest. This review describes the expression patterns of ERα and ERβ in AML and discusses the differing signaling pathways associated with each of these receptors. Furthermore, we assess how these signaling pathways can be targeted by various selective estrogen receptor modulators to induce AML cell death. We also provide insight into ER targeting in AML and discuss pending questions that require further study.

## 1. Introduction

Acute myeloid leukemia (AML) is the most common form of acute leukemia in adults, accounting for approximately 21,000 new diagnoses annually in the U.S. [1]. It is an aggressive hematological malignancy characterized by the accumulation of immature myeloid cells in the blood and bone marrow that impairs normal hematological and immune functions [2]. During hematopoiesis, mature blood cells with specific but varying functions are created through a highly regulated and hierarchal process originating from a self-renewing hematopoietic stem cell (HSC) (i.e., through differentiation) [3]. Similarly, AML is initiated from a rare population of cells known as leukemia stem cells (LSCs), which are quiescent and resistant to typical chemotherapeutics that target mitotic replication [4].

AML can result from an accumulation of genetic mutations, chromosomal translocations and/or epigenetic modifications that occur in numerous combinations and are unique from patient to patient [5]. This variability in disease pathology results in limited therapy options and poor patient outcomes. In fact, the most common treatment for AML is the “7 + 3” regimen of the DNA-targeting drugs cytarabine and daunorubicin, which has remained essentially unchanged for almost 50 years [6]. Furthermore, this regimen is unselective and often intolerable in older patients (>60 years of age), resulting in a 5-year survival rate of only 5%–10% in this population [7]. As such, investigations into novel anti-AML drug-able targets are highly needed.

Estrogen signaling occurs through two estrogen receptors (ERs): ERα and ERβ, which are encoded by the ESR1 and ESR2 genes, respectively [8]. Although ERα was previously thought to be the only ER, the discovery of ERβ in 1996 showed that ERs can have unique functions and varied tissue distributions [9,10]. Generally, ERα exerts a pro-growth effect and is highly expressed in pituitary, vaginal and uterine tissues [11,12]. In contrast, ERβ has anti-proliferative effects and is largely expressed in bone marrow stem cells and lung, colon, mammary gland and prostate tissues [13,14,15,16,17]. ERs regulate transcription through the recruitment of transcriptional coregulators that act as either coactivators or corepressors of genes [18]. Only a subset of these coregulators are common between the two receptor subtypes, which may contribute to their distinct actions [19,20]. The various roles of estrogens have also been demonstrated in several tumor types. In a subset of breast and prostate cancers, ERα expression increases with disease progression, whereestradiol (E2), the principal estrogen hormone, produces tumorigenic effects. By contrast, in colon cancer, where ERβ is predominantly expressed, estradiol has protective effects against malignant transformation [18].

Although AML is not a sex-hormone-related disease, interest in the possible involvement of estrogen and estrogen receptors in AML has stemmed from several epidemiological studies showing sex differences in the incidence of hematological malignancies. Men are approximately two times more likely to be diagnosed with acute or chronic lymphocytic leukemia and various lymphomas, suggesting that estrogen may play a protective role in the development of these diseases [21,22]. Differential expression and ER function in normal and aberrant hematopoiesis have since been studied to better characterize estrogen involvement in disease etiology and progression. In addition, selective estrogen receptor modulators (SERMs), a class of compounds that interact with ERs, have been studied for their effects in treating AML. This review summarizes these findings and provides an overview of the preclinical success of various SERMS.

## 2. ERs in Hematopoiesis

Estrogen signaling has emerged as an important factor in hematopoiesis within the last decade. Sex differences in HSCs show a direct role of estrogen in HSC maintenance and growth. In ovariectomized female rats, the number of HSCs and colony forming units of bone marrow cells are significantly reduced, which suggests that estrogen is required for normal hematopoiesis [23]. Similarly, a study in mice showed that HSCs divide more frequently in females than in males and that exogenous administration of estradiol increased HSC numbers in both males and females [24]. Furthermore, increased HSC numbers occur even without increased bone mass, suggesting that estrogen impacts HSCs independently of its actions on bone [25]. These effects are likely to be mediated through ERα and not ERβ, as this phenotype was lost in ESR1 (ESR1^−/−^) but not in ESR2 (ESR2^−/−^) knock-out mice [25]. Indeed, the ERα activation of HSCs results in the activation of c-myc, the upregulation of its related target genes and a downregulation of c-kit. These transcription factors are highly involved in hematopoiesis and control balance between HSC self-renewal and differentiation [26,27,28]. Not surprisingly, the modulation of c-kit and c-myc was accompanied by increased HSC cell-cycling and a decreased self-renewal capacity of long-term HSCs (LT-HSC) [25,29]. By contrast, multipotent progenitors (MPP) become apoptotic upon ERα activation, suggesting a differential effect of estrogen signaling in hematopoietic cells that may depend on the proliferative capacity or state of differentiation [29]. The role for ERα but not ERβ in HSCs may result from the low-to-non-existent expression of ERβ in murine HSCs, in which the majority of studies were conducted. As such, further insights can be obtained from human-derived HSCs, which express both ER subtypes [30]. In one study using human-derived pluripotent stem cells (hPSCs) and umbilical cord blood cells (UCBs), E2 improved hPSC differentiation in an ERα-dependent pathway, as these effects were not replicated when a specific ERβ agonist was used [31]. Both ERs are expressed in B cells, T cells, NK cells, dendritic cells, erythrocytes and megakaryocytes, suggesting a possible role for ERs in regulating these cell types [32,33,34]. In fact, via ERα, E2 increases the transcription of the interferon regulatory factor 4 (IRF4) to promote dendritic cell differentiation [35,36]. Additionally, ERα but not ERβ activation caused increased erythropoiesis in mice, which is attributed to ERα binding and inhibiting GATA-1, an erythroid transcription factor whose expression increases with erythroid maturation [24,37]. GATA-1 inhibition induced the apoptosis of erythroid cells; however, this was offset by the increased generation of megakaryocyte-erythroid progenitors (MEPs) [24]. By contrast, in megakaryocytes, binding of ERβ to GATA-1 results in increased transcription, which is necessary to promote megakaryocyte polyploidization and platelet formation [38].

**Figure 1:**
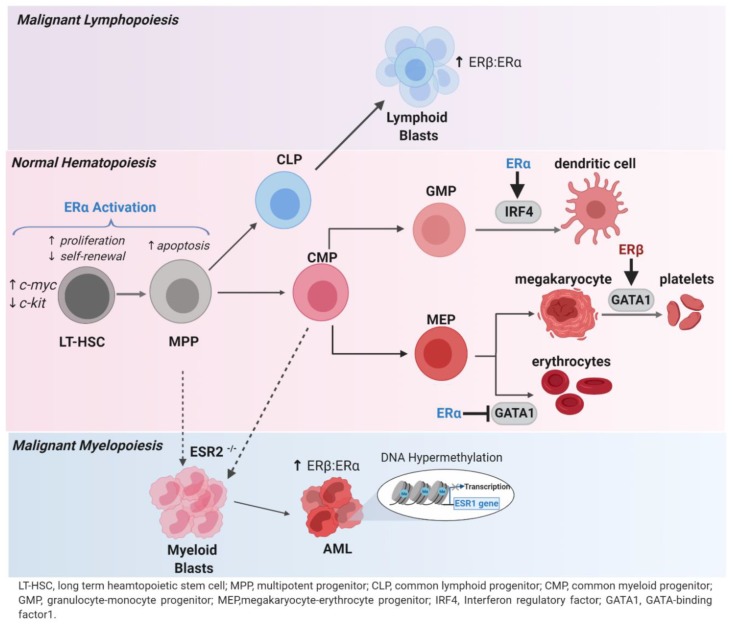
The role of estrogen receptors in hematopoiesis and acute myeloid leukemia (AML). In hematopoiesis, the activation of ERα by estradiol or tamoxifen increased proliferation and decreased self-renewal of primitive long-term hematopoietic stem cells (LT-HSC). The upregulation of c-myc and downregulation of c-kit upon ERα activation contributes to this phenotype. Multipotent progenitors (MPP) undergo apoptosis while ERα-mediated activation of IRF4 encourages dendritic cell differentiation. Additionally, ERα-mediated inhibition of GATA1 increases erythropoiesis while the ERβ-mediated activation of GATA1 results in increased megakaryocyte polyploidization to produce platelets. In malignancy, lymphoid-related cancers predominantly express the ERβ subtype and its activation inhibits tumor growth. The knockdown of ESR2, the gene encoding ERβ, leads to a myeloproliferative disease in mice resembling chronic myeloid leukemia. The possible origins of this disease are unknown and thus symbolized by dashed arrows. In AML, the DNA hypermethylation of ESR1 is prominent among patients and results in decreased transcription of ERα. ERα hypermethylation often co-occurs with the methylation of other tumor suppressor genes, which also influences patient prognosis and the response to hypomethylating agents. A subset of AML patients also express increased ERβ compared to ERα, and it has been shown that this subset would best be targeted by an ERβ agonist.

These studies, summarized in Figure 1, show that although ERα is largely involved in regulating proliferation and self-renewal of HSCs emerging evidence suggests that both ERα and ERβ may be involved in regulating transcription factors associated with differentiation. Nevertheless, ERs play an important role in regulating physiological immune cell function and HSC maintenance; therefore, it is of interest to understand whether ERs are also involved in pathophysiological processes like AML. 

## 3. Estrogen Receptor-α in AML

ERα is expressed in a subset of patient-derived AML cells [39,40]. The relationship between ERα and AML first came to light in 1996, when researchers discovered that the ERα CpG island is aberrantly methylated in 86% of all hematopoietic neoplasms and 91% of AML samples (Figure 1) [41]. Since then, numerous studies with different patient cohorts have corroborated this phenomenon [42,43,44]. ERα methylation (ERM) is most commonly observed in normal karyotype AML and leads to repressed ERα gene transcription [45]. Although frequently observed, there is no consensus regarding the effects of ERM on overall survival and prognosis. One study showed that ERα hypermethylation improved AML survival rates [42], while more recent studies have found no correlation [45] or that ERM worsens survival [46]. ERM was also observed in samples from patients in clinical remission, where increased methylation was associated with a higher risk of relapse [44]. Conclusions regarding ERM and survival are likely to be confounded because ERM frequently co-occurs with epigenetic modifications of tumor suppressor genes involved in various pathways regulating cell growth [47]. However, despite the discrepancies in ERM and overall survival, a relationship between ERM and age has been consistently demonstrated in AML [42]. In contrast to other cancers, ERM is inversely correlated with aging suggesting that methylation may be a result of certain carcinogenic insults rather than an accumulation thereof [42]. Interestingly, of the 54 genes commonly associated with driving AML, 35% have shown evidence of being regulated by ERα and E2 (compared to 65% in breast cancer) [48]. This evidence suggests that aberrations in ERα are commonly found in AML patients but, unlike in breast cancer, are not solely responsible for initiating and maintaining disease. The unique interplay between ERM and other epigenetic modifications must be considered when interpreting data related to ERM and AML. The treatment of hypermethylation in AML is achieved with demethylating agents like azacytidine but has only resulted in mild success. However, predictors of a poor response include the co-occurrence of methylated pro-apoptotic and cell cycle genes [49]. These results show that ERM status alone cannot be used to inform treatment regimens.

## 4. Estrogen Receptor-β in AML

ERβ activation results in pro-death signaling in solid tumors [50,51]. It was also shown to be the predominant ER in lymphoma where its activation strongly inhibited tumor growth [52]. In AML, ERβ is more highly expressed than ERα in some AML patient gene sets [53,54]. Furthermore, we found a subset of AML patients (TCGA) with an increased ERβ/ERα ratio [40]. This, taken together with the prevalent ERα methylation in AML samples, may suggest that ERβ is more predominant in AML. Despite this and recent advances in characterizing the effects of ERβ in normal hematopoiesis, there are many questions about the role of ERβ in AML. Disruption of the ESR2 gene in mice (ERβ^−/−^) resulted in hypercellularity of the bone marrow and myeloproliferative neoplasia resembling chronic myeloid leukemia (Figure 1) [17]. A subsequent study by our group showed that high ERβ/ERα ratios were necessary for the anti-leukemic effects of ERβ signaling (discussed further below) [40]. Although data relating to ERβ in AML are limited, ERβ agonists have shown preclinical effectiveness in solid tumors [55,56,57,58]. In fact, studies in other tumor types with similar ER expression patterns provide further insight into ERβ’s actions in malignancy. In colon cancer, ERβ is predominantly expressed and a positive correlation between this expression and apoptosis is observed [59,60]. ERβ-mediated apoptosis co-occurs with increased DNA fragmentation, increased p53 signaling and the repression of c-myc related pathways [61,62,63]. Additionally, in prostate and bladder cancers, ERβ represses NFκB activation and decreases downstream targets such as the antiapoptotic protein, BCL-2 [64,65]. Interestingly, the aberrant amplification of both c-myc and NFκB occurs in AML; therefore, whether ERβ targeting in AML involves these pathways should be studied further [66,67]. These studies provide encouraging evidence to support future investigations into the anti-leukemic potential of ERβ agonists in AML.

## 5. AML and Selective Estrogen Receptor Modulators (SERMs)

### 5.1. Tamoxifen

Tamoxifen is a SERM with tissue-specific agonist and antagonist activities. Tamoxifen is an ERα antagonist and ERβ agonist in the breast where its antiestrogenic activity is used to treat ERα-positive breast cancer [68]. By contrast, tamoxifen is an ER agonist in other tissues such as the endometrium, bone and, notably, HSCs [69]. In HSCs, the tamoxifen-mediated activation of ER signaling results in proliferation and differentiation in an ERα-dependent manner [29]. Moreover, tamoxifen induces apoptosis in short-term HSCs and MPPs in mice with no effect on their hematological parameters [29], which suggests a differential effect of ERα activation in hematopoiesis whereby primitive cells become more proliferative and less primitive cells become apoptotic. The authors hypothesized that this differential effect could perhaps be exploited in hematological malignancies and subsequently tested tamoxifen on a mouse model of myeloproliferative neoplasm (MPN). Here, treatment with tamoxifen in Janus Kinase 2 (JAK2) V617F mutation-induced MPN caused a significant reduction in disease development and eliminated all of the commonly associated phenotypes of the disease including erythrocytosis, thrombocytosis and leukocytosis [29]. Similarly, in an MLL-AF9 mouse model of AML, tamoxifen improved sensitivity to doxycycline and delayed the reappearance of circulating leukemia cells after chemotherapy [29,70]. In both models, tamoxifen induced apoptosis in malignant cells or restored the levels of HSCs to normal [70]. Interestingly, tamoxifen-induced AML cell apoptosis was preceded by a decrease in mitochondrial respiration and spare reserve capacity, which are directly linked to AML cell survival [71,72]. Despite this, tamoxifen’s pro-apoptotic effects are likely derived from a combination of both ER signaling and metabolic targeting since its effects on HSCs are ERα-dependent. Nevertheless, studies with tamoxifen in ESR1^-/-^ leukemia cells should be conducted to confirm this hypothesis. In another study, tamoxifen and the sphingolipid, C6-ceramide, synergistically induced apoptosis in AML cells and primary samples while sparing normal peripheral blood mononuclear cells (PBMCs) [73]. The combination was preceded by the inhibition of complex I mediated mitochondrial respiration and decreases in ATP synthesis and mitochondrial membrane polarization. Tamoxifen is thought to enhance C6-ceramide cytotoxicity by suppressing its drug metabolism, thereby enhancing its anti-tumor effect [74]. Tamoxifen also improves the all-trans-retinoic acid-induced differentiation of acute promyelocytic HL-60 cells [75] through an ER-related mechanism [76]. These findings, summarized in Table 1, provide support for further studies on the anti-leukemic potential of tamoxifen with an emphasis on characterizing the interplay between ERα and metabolic targeting.

### 5.2. Diosmetin

Diosmetin is a member of the phytoestrogens, which are secondary plant metabolites that are structurally and functionally similar to mammalian estrogen and thus exert estrogenic activity [88]. The anti-leukemic action of diosmetin was demonstrated by our lab using in vitro and in vivo models of AML. We found that it was an ERβ agonist and selectively targeted leukemia blasts and stem cells [40]. Diosmetin’s activity was mediated by ERβ expression, as patient-derived AML cells and cell lines with low levels of ERβ were insensitive to treatment. Furthermore, knockdown of ERβ rendered cells insensitive to diosmetin while ERβ-inducible cells became sensitive to diosmetin upon the induction of ERβ expression [40]. 

Diosmetin inhibited the primary and secondary engraftment of patient-derived AML cells with no effect on normal CD34+ cell engraftment [40]. ERβ activation by diosmetin led to an intracellular accumulation of the pro-apoptotic cytokine TNFα that then resulted in caspase 8 activation and initiation of the extrinsic death receptor-mediated apoptotic pathway [77]. Earlier studies showed that ERβ-expressing monocytes similarly exhibit a TNFα-mediated activation of extrinsic apoptosis that is lost during differentiation to macrophages, which only express ERα [89]. We comparably found that ERβ expression is required for TNFα accumulation and that TNFα mediates diosmetin-induced anti-leukemic activity, as co-incubation with a TNFα-neutralizing antibody abrogated its effects [77]. Taken together, these studies (Table 1) highlight a mechanism by which ERβ activation by diosmetin leads to the selective apoptosis of leukemia cells. Clinical studies in AML patients with high ERβ and low ERα expression are warranted and would provide greater evidence for the potential of ERβ targeting in AML.

### 5.3. Genistein

Genistein is a phytoestrogen found mainly in soy [90]. A common hypothesis is that it contributes to the lower incidence of breast and prostate cancers in Asian countries, due to their high consumption of soy products [91,92]. At a molecular level, it interacts with both ERα and ERβ but is reported to have a greater affinity for ERβ [20]. Genistein exhibits anticancer effects in vitro and in vivo in models of breast [93], colon [94], lung [95], liver [96] and stomach [97] cancer, where it targets ERs, several tyrosine kinases and pro-apoptotic factors [98]. In AML, preclinical studies have shown that it induces cell death in a variety of leukemia cell lines and that a genistein-rich diet improves survival in leukemia-bearing mice [78]. Mechanistically, it has been shown to cause G2/M cell cycle arrest, increase reactive oxygen species (ROS) generation, cause mitochondrial membrane polarization, reduce protein synthesis through inhibition of mTOR, and change the BAX/Bcl2 ratio, leading to apoptosis [79,80,81]. Additionally, genistein and other polyphenols can enhance the anti-AML action of the glycolytic inhibitors 2-deoxy-D-glucose and lonidamine by inhibiting the compensatory activation of protein kinases such as Akt and extracellular signal-regulated kinase (ERK) [82]. Although these findings (summarized in Table 1) provide encouraging evidence for genistein as an anti-leukemia agent, further studies characterizing the functional importance of ERs in these mechanisms of cell death are needed.

### 5.4. Quercetin

Quercetin is a flavanol with estrogenic activity that naturally occurs in many fruits and vegetables [99]. Similar to genistein, quercetin is an agonist for both ERα and ERβ but has a greater affinity for ERβ [100,101]. With these properties, a low ERβ/ERα ratio is necessary for quercetin to confer an anti-proliferative effect, which has been demonstrated in many cancer types to occur through the activation of apoptosis-related mechanisms (i.e., BAX translocation and caspase activation; see Rauf et al. for a comprehensive review) [102]. Studies done in the early 1990s first showed the potential of quercetin to inhibit leukemia cell growth. In patient-derived AML cells, quercetin had a high affinity for a type II ER binding site (i.e., prior to the discovery of ERβ, this was thought to be a site of preferential ER binding for phytoestrogens over estradiol) and a direct correlation between binding to this site and reduced AML cell proliferation was found [39]. They subsequently found that quercetin inhibited the clonogenic growth of patient-derived AML samples with little effect in normal bone marrow samples [83]. Interestingly, these normal bone marrow cells became sensitive to quercetin when the CD34+ fraction was removed, suggesting that hematopoietic progenitors are insensitive to quercetin [83,84]. More recent studies have provided greater insight into the modes of action for leukemia targeting (Table 1). In AML cell lines and tumor xenografts, quercetin reduced tumor growth through ROS-dependent apoptosis that was preceded by poly (ADP-ribose) polymerase (PARP) cleavage and the activation of caspases 3, 8 and 9 [85]. This effect was partly mediated by ERK activation, as co-incubation with an ERK inhibitor partially abrogated quercetin-induced apoptosis [85]. A recent study also showed that the enhancement of apoptosis by quercetin may partly be caused by the inactivation of DNA methylases resulting from the proteasomal degradation of class I histone deacetylases (HDACs), which leads to increased histone acetylation in the promotor regions of pro-apoptotic genes [86]. In particular, this led to the increased transcription of DAPK1, BCL2L11, BAX, APAF1, BNIP3 and BNIP3L, which promoted AML cell apoptosis [86]. In another study, quercetin sensitized AML cells to TNF-related apoptosis-inducing ligand (TRAIL), which activates extrinsic death-receptor mediated apoptosis [87]. The authors of this study report that the TRAIL sensitization by quercetin was a result of the upregulation of death receptor genes DR4 and DR5 and the reduced expression of p65 and the anti-apoptotic proteins XIAP, c-IAP1 and c-IAP2 [87]. While the authors did not correlate these findings with ER activation and expression, this report echoes findings from our lab in relation to the diosmetin-induced activation of ERβ and TNFα-mediated cell death. Collectively, a likely mechanism of activity is through quercetin-induced transcriptional changes following quercetin’s activation of ERβ; however, functional studies to better characterize the role of ERs in quercetin-induced AML cell apoptosis are needed.

## 6. Conclusions

AML is an aggressive disease that requires new drug targets and therapeutic options. Although not regarded as a sex-hormone-related disease, epidemiological and preclinical studies provide compelling data to support ER targeting in AML. Studies have shown that ERα activation enhances the efficacy of conventional chemotherapeutics and that ERβ suppresses leukemogenesis and reduces leukemia cell growth.

In considering ER targeting as an anti-leukemia strategy, it is imperative that the ERβ/ERα ratio in the tested population is known and that the binding of the ER modulator is well defined. Since ERα and ERβ exert opposing actions, ideal drug candidates should be subtype-specific. Furthermore, current evidence provides greater support for specific ERβ agonists as ideal drug candidates, given their anti-proliferative effects in several solid tumors and in AML. Phytoestrogens are of great interest in targeting ERβ, as many of them have demonstrated preferential binding to ERβ over ERα [103]. Synthetic ERβ-specific agonists, like diarylpropionitrile, have demonstrated efficacy in lymphoma but have not yet been studied in AML [52].

Mechanistic studies show that ER modulators activate apoptotic mechanisms, cause mitochondrial dysfunction, or modulate protein kinase signaling and result in cell death. While most studies are correlative, few have demonstrated the functional importance of ER signaling to mitochondrial dysfunction and apoptosis [70,77]. Given that these processes are often deregulated in AML [104,105,106], it would be of great interest to further characterize the importance of ER signaling in this devastating disease. With this, unique treatment regimens with ERβ agonists alone or in combination with apoptotic and/or metabolic modulators could be investigated for their potential to improve AML patient outcome.

## Figures and Tables

**Table 1:** AML and Specific Estrogen Receptor Modulators (SERMs).

Agent	Model(s)	Summary of Results and Underlying Mechanisms	Reference
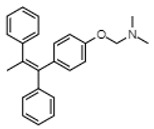 **Tamoxifen**	In vivo: MLL-AF9+ induced AML	Enhanced doxycycline-induced apoptosis, which was preceded by a decrease in mitochondrial respiration and spare reserve capacity.	[29]
	In vitro: HL-60, KG-1 cells, primary AML cells	In synergy with C6-ceraminde, inhibited complex I respiration and induced apoptosis in AML.	[73]
	In vitro: HL-60 cells, primary APL cells	Enhanced ATRA-induced differentiation of APL cells.	[75]
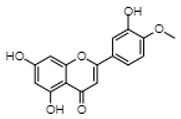 **Diosmetin**	In vitro: TEX, primary AML cells In vivo: Intravenous engraftment of primary AML cells	Reduced the leukemia burden in vitro and in vivo through the targeting of ERβ. High ERβ:ERα ratios were necessary for diosmetin-induced activity.	[40]
	In vitro: TEX, primary AML cells In vivo: Subcutaneous xenograft of TEX cells	ERβ activation by diosmetin increased intracellular TNFα, which activated the extrinsic apoptosis pathway. TNFα increases are lost when ERβ is not expressed. Apoptosis is abrogated by a TNFα-neutralizing antibody.	[77]
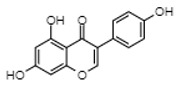 **Genistein**	In vitro: HL-60, MOLT-2, KG1a, Raji cells In vivo: Intravenous engraftment of murine L1210 cells	Inhibited the growth and clonogenicity of myeloid and lymphoid leukemic cell lines, and a genistein-rich diet improved the survival of leukemia bearing mice. It also caused the re-expression of the silenced tumor suppressor genes, p57KIP2 and p15CDKN2B.	[78]
	In vitro: HL-60, MV4-11	Induced caspase-dependent apoptosis of leukemia cell lines and decreased protein synthesis through the inhibition of mTOR. It also inhibited FLT3.	[79]
	In vitro: HL-60 cells In vitro: Subcutaneous xenograft of HL-60 cells	Induced G2/M cell cycle arrest and activated intrinsic and calpain-mediated apoptosis. It also reduced the HL-60 tumor burden.	[80]
	In vitro: U937, Jurkat, K562 cells	Caused G2/M cell cycle arrest and reduced expression of ANXA1, leading to intrinsic apoptosis via caspase 9 activation.	[81]
	In vitro: HL-60, THP-1, NB4 cells	Sensitized AML cells to 2-DG and lonidamine cytotoxicity by inhibiting compensatory Akt and ERK activation, enhancing apoptosis.	[82]
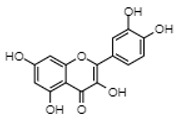 **Quercetin**	In vitro: Primary AML and ALL cells	Primary AML samples expressed a type II estrogen receptor binding receptor for which quercetin had affinity. Quercetin reduced leukemic blast proliferation and inhibited the clonogenic growth of primary AML samples but not CD34+ normal bone-marrow derived cells.	[39,83,84]
	In vivo: HL-60, THP-1, MV4-11, and U937 cell In vivo: Subcutaneous xenograft of HL-60 cells	Caused the ROS-mediated activation of ERK and caspase-mediated apoptosis. It delayed tumor growth in vivo in an ROS-dependent manner, as growth inhibitory effects were lost when mice were co-treated with N-acetylcysteine.	[85]
	In vitro: HL-60, U937 cells In vivo: Subcutaneous xenograft of HL-60 and U937 cells	Abrogated DNMT1 and DNMT3a expression and increased the proteasome degradation of class I HDACs in cell lines and xenograft tumors. It increased the apoptosis of leukemic cell lines by inducing demethylation and the transcriptional activation of pro-apoptotic proteins.	[86]
	In vitro: KG-1 cells	Improved the efficacy of TRAIL in apoptosis induction by upregulating the expression of DR4 and DR5 and downregulating several antiapoptotic proteins like XIAP, c-IAP1 and c-IAP2.	[87]

AML, acute myeloid leukemia; APL, acute promyelocytic leukemia; ATRA, all-trans retinoic acid; ERβ, estrogen receptor beta; TNFα, tumor necrosis factor alpha; mTOR, mammalian target of rapamycin; FLT3, fms like tyrosine kinase 3; ANXA1, Annexin A1; 2-DG, 2-deoxy-D-glucose; ERK, extracellular signal-regulated kinase; DNMT, DNA methyltransferase; HDAC, histone deacetylase; ROS, reactive oxygen species; TRAIL, TNF-related apoptosis-inducing ligand; DR, death receptor; XIAP, X-linked inhibitor of apoptosis protein; c-IAP, cellular inhibitor of apoptosis.

## Abbreviations

AML	Acute myeloid leukemia
APAF1	Apoptotic peptidase activating factor
BAX	Bcl-2-associated X protein
BCL2	B-cell lymphoma 2
BNIP	BCL2 Interacting Protein
c-IAP	Cellular inhibitor of apoptosis
DAPK1	Death-associated protein kinase 1
DR	Death receptor
E2	Estradiol
ER	Estrogen receptor
ERK	Extracellular signal-regulated kinase
ERM	Estrogen receptor alpha methylation
GATA-1	GATA-binding factor 1
HDAC	Histone deacetylase
hPSC	Human pluripotent stem cell
HSC	Hematopoietic stem cell
IRF4	Interferon regulatory factor 4
JAK2	Janus kinase 2
LSC	Leukemia stem cell
LT-HSC	Long term hematopoietic stem cell
MPN	Myeloproliferative neoplasm
MPP	Multipotent progenitor
mTOR	Mammalian target of rapamycin
NFκB	Nuclear factor kappa B
PARP	Poly (ADP-ribose) polymerase
PBMC	Peripheral blood mononuclear cell
ROS	Reactive oxygen species
SERM	Selective estrogen receptor modulator
SMAC	Second mitochondria-derived activator of caspase
TCGA	The Cancer Genome Atlas
TRAIL	TNF-related apoptosis-inducing ligand
UCB	Umbilical cord blood cells
XIAP	X-linked inhibitor of apoptosis protein
